# A critical appraisal of the quality of head and neck cancer imaging guidelines using the AGREE II tool: A EuroAIM initiative

**DOI:** 10.1002/cam4.1933

**Published:** 2018-12-21

**Authors:** Valeria Romeo, Arnaldo Stanzione, Sirio Cocozza, Lorenzo Ugga, Renato Cuocolo, Arturo Brunetti, Sotirios Bisdas

**Affiliations:** ^1^ Department of Advanced Biomedical Sciences University of Naples “Federico II” Napoli Italy; ^2^ Department of Neuroradiology, The National Hospital for Neurology and Neurosurgery University College London NHS Foundation Trust London UK; ^3^ Department of Brain Repair and Rehabilitation, Institute of Neurology University College London London UK

**Keywords:** AGREE II, evidence‐based medicine, guidelines, head and neck cancer, imaging

## Abstract

**Background:**

Diagnostic imaging guidelines are increasingly embraced in oncologic imaging in order to improve examinations appropriateness and technical quality. The usefulness of guidelines employment in clinical practice is dramatically related to the quality of the guidelines themselves. However, an extreme variability in guidelines’ quality may occur. Following a European Network for the Assessment of Imaging in Medicine (EuroAIM) initiative, the aim of this study was to assess the quality of the available guidelines regarding head and neck cancer (HNC) imaging.

**Methods:**

A literature search was conducted to identify imaging guidelines focused on HNC. Selected guidelines were evaluated by four independent appraisers using the Appraisal of Guidelines for Research & Evaluation version 2.0 (AGREE II) tool, which comprises 23 key items, rated on a 7‐point scale (1—strongly disagree to 7—strongly agree) and organized within six domains. For each domain, the intraclass correlation coefficient (ICC) was used to assess the agreement among appraisers’ scores.

**Results:**

After literature search, three guidelines were selected and evaluated. One guideline scored “average” as overall quality, while the remaining two scored a “low” overall quality. The highest result (total score = 75.0% ± 19.3%) was obtained in domain 4 (Clarity of presentation) while the lowest (total score = 27.1% ± 4.2%) in domain 6 (Editorial independence). ICC analysis showed a very good agreement (range: 0.932‐0.961) among the four appraisers.

**Conclusions:**

Our results showed a heterogeneous quality of existing guidelines in HNC imaging. Issues raised from this appraisal should be considered when developing future guidelines on HNC imaging.

## 1 INTRODUCTION

Head and neck cancer (HNC) includes a broad spectrum of malignancies, anatomically related but different in terms of management, accounting for about 4% of cancer incidence in Europe.^1^ Imaging plays a crucial role in the diagnostic evaluation of patients affected by HNC, being able to add pivotal information related to diagnosis, staging, and response to therapy, significantly contributing to improve HNC prognosis.^2^, ^3^, ^4^ Advanced cross‐sectional imaging modalities enable radiologists to assess disease extent, its spread to neighboring structures and local lymph nodes, perineural or perivascular spread, and bone invasion, as well as to identify distant metastases and the presence of relevant comorbidities. Imaging is also used to guide biopsies, for treatment planning (radiotherapy and computer‐assisted surgery), and patient restaging after therapy, while detecting persistent or recurrent disease; moreover, it has been proved to increase the diagnostic yield in the unknown head and neck primary population, allowing a more targeted treatment.^5^

Therefore, an appropriate use of the available imaging techniques appears of paramount importance to achieve the best outcome for patients. For this reason, diagnostic imaging guidelines for physicians and radiologists have been released with the aim of improving appropriateness and technical quality of imaging examinations.^6^, ^7^, ^8^, ^9^, ^10^ While guidelines are increasingly embraced in oncologic imaging, concerns have been raised about their reliability since their quality can be remarkably variable, which dictates their methodological evaluation^11^, ^12^ and may potentially affect guideline usefulness and benefits.

Among various quality appraisal instruments developed for evaluating guidelines quality in terms of methodological rigor and transparency,^13^ the Appraisal of Guidelines for Research & Evaluation version 2.0 (AGREE II) has been validated and proved to be a reliable tool^14^ being already applied for clinical practice guidelines in different fields, including HNC.^15^, ^16^, ^17^, ^18^, ^19^ In this setting, the European Network for the Assessment of Imaging in Medicine (EuroAIM) was founded by the European Institute for Biomedical Imaging Research (EIBIR)^20^ with the purpose of seeking evidence for the best use of radiological technology. Recently, EuroAIM has focused its activity on the evaluation of the quality of guidelines in different fields of diagnostic imaging.^21^, ^22^ Considering the well‐conducted previous experiences focused on specific organs belonging to the head and neck area (eg, thyroid cancer),^17^, ^19^ we decided to embrace a wider approach focusing our analysis on guidelines dealing with head and neck in a more comprehensive view. Therefore, the aim of this study was to evaluate the quality of current guidelines on imaging of HNC using the AGREE II quality assessment tool.

## 2 MATERIALS AND METHODS

### 2.1 Literature search and guidelines selection

Between March and April 2018, a literature search was conducted on PubMed, EMBASE, Web of Science, Scopus, Wiley Online Library, and Google to identify imaging guidelines focused on HNC, using the following keywords: “head and neck cancer imaging,” “head and neck neoplasms,” “guidelines,” “recommendations,” “official positions,” and their expansions. The reference section of the retrieved papers was also checked in order to seek further articles to include. Inclusion criteria were the following: (a) guidelines focused on HNC imaging; (b) guidelines concerning adult population; and (c) guidelines with available English full text. Exclusion criteria were as follows: (a) guidelines not exclusively or primarily focused on HNC imaging (eg, clinical practice guidelines in which imaging is described in a wider context); (b) guidelines not developed by recognized institutions/groups of affiliated governmental organizations; and (c) guidelines not including all major imaging techniques (ie, ultrasound, computed tomography, magnetic resonance imaging, positron emission tomography). In case of disagreement among the appraisers during the screening process, inclusion and exclusion criteria were evaluated and applied in consensus.

### 2.2 AGREE II tool

Guidelines were evaluated using the AGREE II tool, which comprises twenty‐three key items organized within six quality domains: domain 1 = “Scope and purpose” (items 1‐3); domain 2 = “Stakeholder involvement” (items 4‐6); domain 3 = “Rigor of development” (items 7‐14); domain 4 = “Clarity of presentation” (items 15‐17); domain 5 = “Applicability” (items 18‐21); and domain 6 = “Editorial independence”(items 22‐23); domains are then followed by two additional items (“Overall Assessment”) to assess “the rating of the overall quality of the guideline and whether the guideline would be recommended for use in practice”.^14^ A comprehensive description of all AGREE II domains and items is reported in Table 1.

**Table 1:** Description of AGREE II domains and items (ref.^14^)

Domain 1. Scope and purpose	Item 1: The overall objective(s) of the guideline is (are) specifically described
	Item 2: The health question(s) covered by the guideline is (are) specifically described
	Item 3: The population (patients, public, etc) to whom the guideline is meant to apply is specifically described
Domain 2. Stakeholder involvement	Item 4: The guideline development group includes individuals from all the relevant professional groups
	Item 5: The views and preferences of the target population (patients, public, etc) have been sought
	Item 6: The target users of the guideline are clearly defined
Domain 3. Rigor of development	Item 7: Systematic methods were used to search for evidence
	Item 8: The criteria for selecting the evidence are clearly described
	Item 9: The strengths and limitations of the body of evidence are clearly described
	Item 10: The methods for formulating the recommendations are clearly described
	Item 11: The health benefits, side effects, and risks have been considered in formulating the recommendations
	Item 12: There is an explicit link between the recommendations and the supporting evidence
	Item 13: The guideline has been externally reviewed by experts prior to its publication
	Item 14: A procedure for updating the guideline is provided
Domain 4. Clarity of presentation	Item 15: The recommendations are specific and unambiguous
	Item 16: The different options for management of the condition or health issue are clearly presented
	Item 17: Key recommendations are easily identifiable
Domain 5. Applicability	Item 18: The guideline describes facilitators and barriers to its application
	Item 19: The guideline provides advice and/or tools on how the recommendations can be put into practice
	Item 20: The potential resource implications of applying the recommendations have been considered
	Item 21: The guideline presents monitoring and/or auditing criteria
Domain 6. Editorial independence	Item 22: The views of the funding body have not influenced the content of the guideline
	Item 23: Competing interests of guideline development group members have been recorded and addressed

### 2.3 Guidelines evaluation

According to AGREE recommendation, four independent appraisers (VR, LU, SC, and RC) with 5‐8 years’ experience in head and neck diagnostic imaging and the field‐related research performed the evaluation, in order to increase the reliability of the assessment. Prior to the evaluation, all appraisers had performed the online training tool, freely available on the AGREE platform (www.agreetrust.org). For each item, a score from 1 to 7 was rated. Score 1 (strongly disagree) was given when there was no relevant information concerning the item, the concept was poorly reported or criteria were not met; scores from 2 to 6 were assigned when the reporting of the AGREE II item did not meet the full criteria or considerations. Score 7 (strongly agree) was assigned if the quality of reporting was exceptional and the full criteria were met. For each domain, final scores were calculated as the sum of individual items scores, expressing the total as a percentage of the maximum possible score for that domain. The overall quality was evaluated using a threshold of 60% for the final score of each domain, with quality defined as “high” when 5 or more domains scored >60%, “average” when 3 or 4 domains scored >60%, and “low” when ≤2 domains scored >60%. In addition, overall scores, expressed as mean ± standard deviation (SD) of both guidelines and domains, were calculated; domain scores were categorized as good (≥80%), acceptable (60%‐79.9%), low (40%‐59.9%), or very low (<40%).

### 2.4 Statistical analysis

To assess the agreement among the four appraisers, intraclass correlation coefficient (ICC) was used and classified as follows: poor (<0.20); fair (0.21‐0.40); moderate (0.41‐0.60); good (0.61‐0.80); and very good (0.81‐1.00), according to previous evidences.^21^, ^22^

Data extraction, collection, and scoring were performed by an independent reviewer (AS) with 5 years of experience in statistical analysis of biomedical research data, using Microsoft Excel^®^ 2016 (Microsoft Corporation, Redmond, WA, USA), while ICC analysis was performed using the Statistical Package for Social Science (SPSS) software (version 24, IBM, Armonk, NY, USA).

## 3 RESULTS

**Figure 1:**
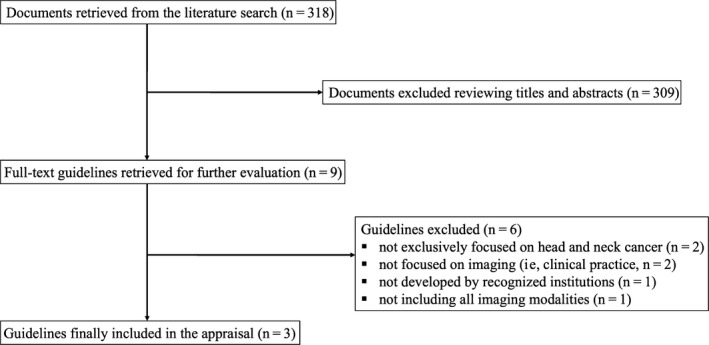
Flow diagram of guidelines selection

After exhaustive literature search, 318 papers were retrieved of which three, published between 2012 and 2016, fulfilled all inclusion and exclusion criteria and were selected for the evaluation. Flow diagram of the selection process is illustrated in Figure 1, and details of the selected papers are reported in Table 2. Of the selected guidelines, one scored “average” as overall quality, with three domains reaching percentage scores >60%, while the remaining two scored a “low” overall quality, with no more than one domain reaching a percentage score >60%. In particular, based on the average score of all domains, the guideline that reached the highest score (55.24%) was the “American College of Radiology, ACR Appropriateness Criteria: neck mass/adenopathy” (ACR),^6^ while the remaining two “Recommendations for cross‐sectional imaging in cancer management, Second edition—Head and neck cancer” by the Royal College of Radiology (RCR)^7^ and “Imaging in head and neck cancer: United Kingdom National Multidisciplinary Guidelines” (UKNMG)^8^ rated lower scores (34.81% and 46.96%, respectively). Regarding overall domains’ scores, the highest (75%) was found for domain 4 “Clarity of presentation” while the lowest (27.1%) for domain 6 “Editorial independence.” The highest variability (SD 20.63%) was found in domain 2 “Stakeholder involvement” and the lowest (4.17%) in domain 6 “Editorial independence.” All average domains’ scores are reported in Table 3. In particular, domain 1 (Scope and purpose) obtained scores of 66.67%, 52.78%, and 59.72% for ACR, RCR, and UKNMG guideline, respectively, with a mean score of 59.72% and a low variability (SD 6.94%). In domain 2 (Stakeholder involvement), the highest variability (SD 20.62%) was found among the three guidelines, due to discrepancies among the assigned scores; in particular, the highest score (61.11%) was assigned to ACR guideline, while the lowest (20.83%) to UKNMG, with an average score of 38.43%. Regarding domain 3 (Rigor of development), the guideline with the highest score was that of the ACR with 56.77%, while the lowest (21.35%) was scored by RCR guideline; due to this discrepancy, a SD of 19.87% was found for this domain with a mean score of 33.85%. In domain 4 (Clarity of presentation), a score of 87.5%, 84.72%, and 52.78% was calculated for ACR, RCR, and UKNMG guidelines, respectively, with a mean value of 75% ± 19.3%. RCR guideline reached the highest (58.33%) score for domain 5 (Applicability), with lower scores assigned to ACR (32.29%) and UKNMG (29.17%), a final average score of 39.93% and a SD of 16%. The lowest variability (SD 4.17%) was found in domain 6, with a more homogeneous scoring of 27.08%, 31.25%, and 22.92% for ACR, RCR, and UKNMG guidelines, respectively, and a mean percentage of 27.08%. ICC analysis showed a very good agreement among the four appraisers; in detail, the ICC was 0.961 (CI 95%: 0.848‐0.994) for ACR guideline, 0.948 (CI 95%: 0.822‐0.992) for RCR guideline, and 0.932 (CI 95%: 0.729‐0.989) for UKNMG.

**Table 2:** Guidelines selected for the evaluation

Title	Country of origin	Year	Organization
American College of Radiology (ACR): Neck mass/adenopathy ACR Appropriateness Criteria^6^	USA	Date of origin: 2009 Last review date: 2012	American College of Radiology (ACR)
Recommendations for cross‐sectional imaging in cancer management, Second edition—Head and neck cancer^7^	UK	2014	The Royal College of Radiology
Imaging in head and neck cancer: United Kingdom National Multidisciplinary Guidelines^8^	UK	2016	British Association of Endocrine and Thyroid Surgeons, British Association of Head and Neck Oncologists, British Association of Oral and Maxillofacial Surgeons, British Association of Otorhinolaryngology‐Head and Neck Surgery, British Association of Plastic, Reconstructive and Aesthetic Surgeons, The Royal College of Pathologists and The Royal College of Radiologists (Faculty of Clinical Oncology)

**Table 3:** Summary of the average domains’ scores of HNC guidelines according to AGREE II

Domain	ACR^6^	RCR^7^	UKNMG^8^	Total score mean	SD	Overall domain score
1. Scope and purpose	66.67	52.78	59.72	59.72	6.94	Low
2. Stakeholder involvement	61.11	33.33	20.83	38.43	20.62	Very low
3. Rigor of development	56.77	21.35	23.44	33.85	19.87	Very low
4. Clarity of presentation	87.50	84.72	52.78	75.00	19.30	Acceptable
5. Applicability	32.29	58.33	29.17	39.93	16.01	Very low
6. Editorial independence	27.08	31.25	22.92	27.08	4.17	Very low
Total score mean	55.24	46.96	34.81			
Overall quality	Average	Low	Low			

ACR, “American College of Radiology, ACR Appropriateness Criteria: neck mass/adenopathy”; RCR, “Recommendations for cross‐sectional imaging in cancer management, Second edition, Head and neck cancer” by the Royal College of Radiology; SD, standard deviation; UKNMG, “Imaging in head and neck cancer: United Kingdom National Multidisciplinary guidelines”; All values are expressed as percentages.

## 4 DISCUSSION

According to the AGREE II tool, we demonstrated a heterogeneous quality of existing guidelines on HNC imaging. One guideline reached an “average” level of quality, while the remaining two scored a “low” level of quality. Domains in which the highest percentage score was obtained were domain 1 “Scope and purpose” and domain 4 “Clarity of presentation” since aims, target users, and key recommendations were clearly specified in all papers; this is in line with results obtained in previous evaluations,^23^, ^24^ probably because these issues are essential for guideline drafting and therefore properly considered. On the basis of AGREE II items, the quality in these domains could be further improved specifying timing for follow‐up, stratifying recommendations on the basis of clinical features (eg, HPV status), or proposing alternative options for the use of different imaging techniques. In the remaining 4 domains, overall scores <40% were obtained. In detail, domain 2 “Stakeholder involvement” obtained an average score of 38.43%, mainly due to the lack of information about target population views and preferences (domain 2), as also addressed by Lin and colleagues regarding the evaluation of guidelines for musculoskeletal pain.^25^ The noninvolvement of relevant professional figures other than radiologists in guideline's draft except for ACR guideline (in which neurologists, surgeons, and nuclear medicine specialists were also involved) could also explain this finding along with the highest variability (SD 20.62%) found among guidelines’ scores in this domain. Domain 3, “Rigor of development,” scored an average percentage score of 33.85% since methods for searching or evaluating evidences were not always specified and no guideline authors’ team applied the Delphi or Glaser technique to achieve a mutual agreement among experts.^26^ However, it should be noted that certain items in this domain (eg, items 12 and 14) may not attract enough attention and, even if not reported, these methodological aspects are often adequately performed.^16^, ^27^, ^28^ Regarding domain 5 “Applicability,” facilitators and barriers for guidelines’ application, resource implications, advice on how the recommendation can be put into practice as well as monitor or auditing criteria were not clearly specified in the guidelines under evaluation, resulting in the rather poor average score of 33.85%. Interestingly, this domain score was also found to be low in previous appraisals of clinical guidelines published in HNC,^15^, ^16^, ^18^ suggesting that the applicability of recommendations is underestimated during guidelines drafting. Finally, as also occurred in previous evaluations,^21^, ^22^, ^29^ domain 6 “Editorial Independence” was the most critically underperforming with an average score of 27.08% and the lowest variability (SD 4.17%), since neither an explicit statement that the funding body interests have not influenced the final recommendations nor “no conflict of interest” statement were provided in two out of three guidelines. This is a crucial issue since the prevalence of conflicts of interest has been proved to be high among members of clinical practice guideline panels.^30^

With regard to the interobserver agreement among the four appraisers in this study, the ICC analysis showed a very good degree of concordance, thus confirming the suitability of AGREE II tool. Its main strength lies in providing clear instructions for its application, enabling users to optimally perform the appraisal after completing the online training session.

According to the AGREE II instructions, it would have been possible to prioritize one domain over the others before beginning the appraisal, based on the evaluation of the importance of the different domains and items in this context. Nevertheless, in agreement with the previous experiences^21^, ^22^ we did not prioritize a specific domain over the others as we consider all quality domains to equally contribute in determining the clinical implications of guidelines while evaluating different guidelines attributes.

Based on this evaluation, possible improvements in future guidelines on HNC imaging should be pursued giving particular attention to the Editorial independence, since guidelines are often developed with external funding or in some circumstances members of the development group may have competing interests introducing hence concealed bias. An acknowledgment section including an explicit statement in which the authors have declared if there are competing interests should be provided. Furthermore, robust, standardized methodologies for evidence research and evaluation (eg, using the Delphi or Glaser method) are warranted to strengthen the reliability of the process and should be reported in dedicated paragraphs. Strengths and limitations of the selected evidence should also be acknowledged and discussed as well as the methodology for guideline external review should be described. The inclusion of “multidisciplinary” groups, composed by different professional stakeholders other than radiologists (eg, radiation, medical, and surgical oncologists), would lead to a dramatic improvement in the HNC guidelines quality. Last but not least, issues such as the availability/costs of the recommended techniques should be considered in order to significantly improve guidelines quality and facilitate their translation into clinical practice. Specifically, it should be noted that the AGREE II tool does not accommodate any parameters related to dissimilarities among the various national healthcare systems as funding sources, patient cost‐sharing, availability and access to the cross‐sectional and hybrid imaging modalities, prevalence and socioeconomic burden of the disease linked with its cure rate and morbidity, and the reimbursement model either per item or per patient. Such improvements might be considered and incrementally included in future expansions and revisions of the AGREE tool.

Some limitations of the present study should be acknowledged. First, the AGREE II tool rates the degree of methodological rigor in guidelines, which is strongly related to but is not an index of the quality of content. Consequently, possible benefits of the recommendations on patients’ management could not be directly assessed. Second, considerable merit is given to the inclusion of specific statements related to possibly implicit issues, which may negatively affect the final score of certain domains despite the elaborated methodological quality. Nevertheless, the AGREE II tool has been extensively validated and its limits are common among different appraisals tools.^13^ Finally, a low number of guidelines were included in this analysis; it should also be noted that since the selection included only guidelines published in English some guidelines might have been missed.

In conclusion, our AGREE II tool‐based analysis showed a heterogeneous quality of the existing guidelines on clinical HNC imaging. Issues raised from this appraisal should be considered when developing future guidelines in the field.

## CONFLICT OF INTEREST

None declared.
